# Rapid, Massive, and Green Synthesis of Polyoxometalate-Based Metal–Organic Frameworks to Fabricate POMOF/PAN Nanofiber Membranes for Selective Filtration of Cationic Dyes

**DOI:** 10.3390/molecules29071493

**Published:** 2024-03-27

**Authors:** Jianping Li, Zhaoke Yu, Jiaming Zhang, Chengjie Liu, Qi Zhang, Hongfei Shi, Dai Wu

**Affiliations:** College of Materials Science and Engineering, Jilin Institute of Chemical Technology, Jilin 132022, China; yu2576289101@163.com (Z.Y.); 13943133446@163.com (J.Z.); L18660052182@163.com (C.L.); 17696079502@163.com (Q.Z.); shihf813@nenu.edu.cn (H.S.)

**Keywords:** polyoxometalates, polyoxometalate−based metal−organic frameworks, green synthesis, membrane separation, dye removal, nanomaterial

## Abstract

Developing high−efficiency membrane materials for the rapid removal of organic dyes is crucial but remains a challenge. Polyoxometalates (POMs) clusters with anionic structures are promising candidates for the removal of cationic dyes via electrostatic interactions. However, their shortcomings, such as their solubility and inability to be mass−produced, hinder their application in water pollution treatment. Here, we propose a simple and green strategy utilizing the room temperature stirring method to mass produce nanoscale polyoxometalate−based metal−organic frameworks (POMOFs) with porous rhomboid−shaped dodecahedral and hexagonal prism structures. The products were labeled as POMOF**1** (POMOF-PW_12_) and POMOF**2** (POMOF-PMo_12_). Subsequently, a series of x wt% POMOF**1**/PAN (x = 0, 3, 5, and 10) nanofiber membranes (NFMs) were prepared using electrospinning technology, where polyacrylonitrile (PAN) acts as a “glue” molecule facilitating the bonding of POMOF**1** nanoparticles. The as−prepared samples were comprehensively characterized and exhibited obvious water stability, as well as rapid selective adsorption filtration performance towards cationic dyes. The 5 wt% POMOF**1**/PAN NFM possessed the highest removal efficiency of 96.7% for RhB, 95.8% for MB, and 86.4% for CV dyes, which realized the selective separation over 95% of positively charged dyes from the mixed solution. The adsorption mechanism was explained using FT−IR, SEM, Zeta potential, and adsorption kinetics model, which proved that separation was determined via electrostatic interaction, hydrogen bonding, and π–π interactions. Moreover, the POMOF**1**/PAN membrane presented an outstanding recoverable and stable removal rate after four cycles. This study provides a new direction for the systematic design and manufacture of membrane separation materials with outstanding properties for contaminant removal.

## 1. Introduction

Organic dyes are frequently used in various industries such as plastics, textiles, inks, coatings, and electroplating. Among these organic dyes, cationic dyes form a crucial subset [1,2]. Unfortunately, some cationic dyes have been shown to display biotoxicity, low biodegradability, and carcinogenic and mutagenic properties, posing risks to both humans and the environment, and may cause various diseases [3,4]. Therefore, it is very important to find appropriate methods and efficient materials in order to eliminate cationic dyes before discharging sewage. Several methods have been proposed and applied, such as ion exchange, oxidation, photocatalysis, membrane filtration, biotechnology, and so on [5,6,7]. Notably, membrane filtration has emerged as a prevalent approach for cationic dye removal due to its ease of recovery, high efficiency, eco−friendliness, and simple operation [8]. However, due to the lack of porous adsorption materials with inherent charges for efficient membrane production, membrane filtration technology still faces substantial challenges, as electrostatic interactions are one of the most effective mechanisms for the adsorption and separation of organic pollutants. This prompted researchers to design novel adsorbent materials with greater negative potential to enhance the affinity and electrostatic attraction for the selective separation of cationic dyes.

As a type of anionic framework material, polyoxometalates (POMs) are composed of transition metals with the highest valence state and oxygen atoms and possess structural diversity, redox properties, potent electron−accepting capabilities, and unique multiple charge properties [9,10,11]. To date, POMs have been widely used in multiple fields such as energy conversion and storage, catalysis, and drug delivery [12,13,14]. Keggin−type heteropolyanions such as phosphotungstic acid (H_3_PW_12_O_40_, *abbr.* PW_12_) and phosphomolybdic acid (H_3_PMo_12_O_40_, *abbr.* PMo_12_) are one of the classical structures of POMs that possess lower spatial resistance, stronger coordination ability, and sub−nanometer−size (~1 nm) [15]. These excellent intrinsic characteristics render them ideal building units for constructing new compounds, and researchers have constructed some POM−based composites that can used to adsorb cationic dyes [16,17]. POM clusters with anionic structures are promising candidates for the removal of cationic dyes using electrostatic interactions. However, in the field of wastewater treatment, the application of POMs in the adsorption and separation of pollutants is hindered due to their small specific surface area, self−aggregation tendency, good solubility in water, and inability to be mass−produced. Consequently, various POM−based composite materials have been devised by amalgamating POMs with different support materials, enhancing the stability and dispersion characteristics of the POMs. Among numerous supporting materials, metal–organic frameworks (MOFs) represent a burgeoning porous crystalline material with inherent porosity, large surface area, adjustable pore size, insolubility, and strong adaptability, which have been widely used in wastewater treatment [18]. However, most MOFs materials are neutral and possess poor affinity relative to specific ionic dyes, which limits their removal rates and selective filtration ability [19]. Based on the above considerations, polyoxometalate–based metal–organic frameworks (POMOFs) are candidates for efficient adsorbents, combining the advantages of both materials and overcoming their respective shortcomings in water treatment. In addition, another obstacle to their practical application in wastewater treatment is that high−efficiency adsorbent POMOFs materials cannot be mass−produced, with the exception of a few, and the recycling of powder material requires a complex recycling process [20,21]. Manufacturing fiber membranes based on POMOFs and the polymer matrix appears to have good prospects.

Electrospinning is a technique employed to manufacture fiber membranes with diameters ranging from nanometers to micrometers. These membranes retain the specific functionalities of the constituent materials and have extensive applications in water purification due to their high porosity, ease of recycling, and straightforward preparation [22,23,24]. Polyacrylonitrile (PAN) is a low−cost polymer that is easy to make from nanofibers through electrospinning. For example, Teng et al. developed a PAN@C/MIL−101(Fe) NFM for tetracycline hydrochloride adsorption in water. They achieved an adsorption capacity of 392.64 mg/g, maintaining 94% efficiency consistently over five cycles [25]. Wang et al. developed bead PAN/ZIF−8 NFM via one-step electrospinning, achieving a maximum adsorption capacity of 224.37 mg/g under conditions of 100 mg/L methyl blue concentration for 180 min [26]. Meanwhile, Wu et al. demonstrated that pMIL−88A/PAN NFM exhibited remarkable dye removal rates of 99.2% for AR, 94.4% for RhB, and 99.8% for AB. Even after five adsorption cycles, the dye elimination rate of pMIL−88A/PAN NFM remained above 80% [27]. Therefore, using electrospinning technology to combine POMOFs with polymers to fabricate nanofiltration membranes could be an effective method.

Following the above considerations, a POMOF/PAN nanofiber membrane (NFM) was successfully prepared. Two POMOF nanomaterials that can be mass−produced were prepared through a green synthesis approach for the first time. Their structural analysis results indicate that POMOF**1** has a porous rhomboid−shaped dodecahedron and POMOF**2** has a hexagonal prism structure possessing isomorphic structures that are formed by encapsulating the negatively charged POMs (PW_12_ or PMo_12_) in the [Co_4_(dpdo)_12_]_∞_ (4,4′−bipyridine-N,N′−dioxide *abbr.* dpdo) framework. In order to ensure its practicality, the powdery POMOF**1** nanomaterial was processed into membranes with PAN polymers using the electrospinning technique and tailored for the filtration of cationic dyes. The incorporation of POMOF nanoparticles into the PAN matrix not only improves the hydrophilicity of the membrane but also plays an important role in enhancing the adsorption performance. The as−prepared POMOF**1**/PAN NFM exhibits good wettability, negative Zeta potential, and effective filtration of cationic dyes under gravity, indicating its potential as a rapid and selective filter for dye separation in aqueous solutions. The filtration efficiencies of RhB, MB, and CV are 96.7%, 95.8%, and 86.4% when using 5 wt% POMOF**1**/PAN NFM. Even after four recycling cycles, the dye removal rate remains consistently high at 90%, indicating excellent reusability and promising practical application prospects. Such a simple and universal manufacturing process is beneficial for constructing filtration membranes with self−supporting and porous characteristics that are significant for the application of wastewater treatment.

## 2. Results and Discussion

### 2.1. Preparation and Characterization of POMOF**1** and POMOF**1**/PAN NFM

**Scheme 1:**
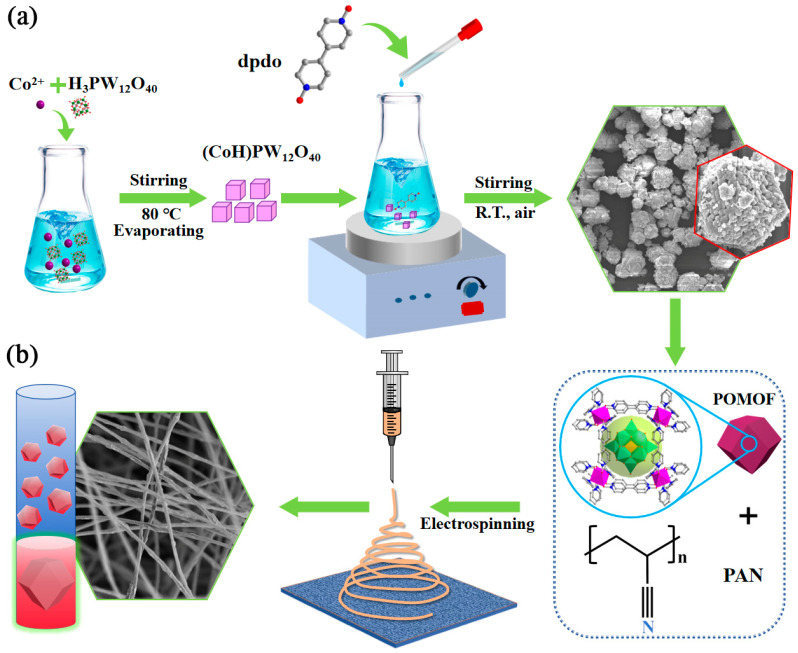
Schematic diagram of the synthesis process of (**a**) POMOF and (**b**) POMOF/PAN NFM.

POMOF**1** and POMOF**1**/PAN NFM were prepared via conventional synthesis and electrospinning technology. As revealed in Scheme 1a, POMOF**1** was synthesized using a modified method [28] that can realize green synthesis with high yield. Briefly, the transition metal cobalt ion (Co^2+^) was used as a counter−ion to replace two H^+^ in H_3_PW_12_O_40_ at 80 °C for 4 h to form (CoH)PW_12_O_40_. Then, the dpdo aqueous solution was gradually added to the (CoH)PW_12_O_40_ solution under stirring at room temperature to synthesize POMOF**1**. (The process of POMOF**2** synthesis was the same as for **1**). POMOF**1**/PAN NFM were also prepared using electrospinning technology. Initially, the as-prepared POMOF**1** and polyacrylonitrile (PAN) were blended in a mixture solution of DMF and acetonitrile to form the spinning solution, and then the composite fiber membrane was generated via electrospinning, as shown in Scheme 1b. PAN acts as a “glue” molecule to promote the bonding between POMOF**1** nanoparticles. The detailed preparation processes are described in Section 3.2 and Section 3.3.

**Figure 1:**
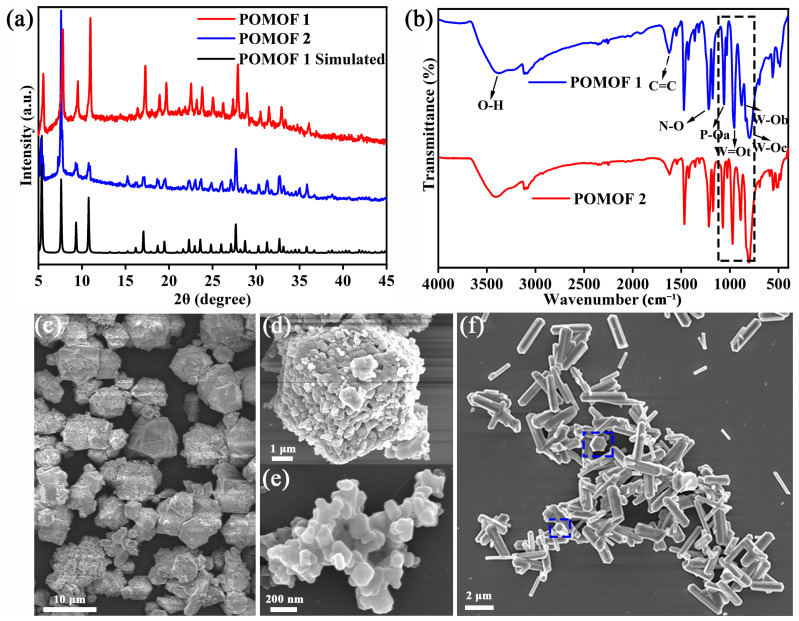
(**a**) The XRD patterns and (**b**) FT−IR spectra of POMOF(**1**, **2**); (**c**–**e**) SEM of POMOF**1** powder; (**f**) SEM of POMOF**2** powder.

The structure, composition, and crystallinity of the synthesized materials were confirmed through powder X−ray diffraction (XRD). As revealed in Figure 1a, POMOF (**1**, **2**) possessed the same diffraction patterns and matched the reported single−crystal data (CCDC: 628313) [29] of simulated POMOF**1** curves. The results indicated that POMOF (**1**, **2**) nanomaterials were successfully synthesized for pure crystalline phase and possessed isomorphic structures that were three-dimensional (3D) (Figure S1). As is well known, water stability is crucial for water treatment materials. Acid–base water stability experiments of the POMOF**1** powder were explored by immersing sample POMOF**1** in solutions with different pH values for 2 h, as shown in Figure S2. The results show that the structure of POMOF**1** remained unchanged in the pH range of 1−10, suggesting excellent stability of POMOF**1**, meaning that it can treat contaminants in broad wastewater pH ranges. The composition and structure of the material can be determined by analyzing the functional groups in the infrared spectrum of a material. Figure 1b shows the Fourier transform infrared spectroscopy (FT−IR) spectra of the two types of POMOFs, which displayed four characteristic vibrations originating from the Keggin structure heteropolyanions: ν(W=Ot) 956, ν(W−Ob) 879, ν(W−Oc) 806, and ν(P−Oa) 1061 cm^−1^, along with ν(Mo=Ot) 976, ν(Mo−Ob) 890, ν(Mo−Oc) 805, and ν(P−Oa) 1079 cm^−1^. Additionally, the IR spectra of the dpdo molecule exhibited four characteristic vibrations: ν(N−O) 1218, ν(ring) 1472, and δ(C−H, in-plane) 1178 cm^−1^, as well as O−H and C−H vibrations (at 3383 and 3113 cm^−1^, respectively). The corresponding functional group at 1640 cm^−1^ is a C=C stretching vibration [28]. The conclusive evidence confirmed the successful synthesis of the POMOF materials.

The morphologies, microstructures, and sizes of the two POMOF(**1**, **2**) materials were observed using scanning electron microscopy (SEM). Figure 1c displays the morphology of POMOF**1**, presenting a porous rhombic dodecahedron with a uniform shape, approximately 8 μm in diameter. Figure 1d,e further depicts the formation process illustrated in Figure 1c. It can be inferred that the porous rhombic dodecahedron was self−assembled from numerous small cubic blocks (around 100 nm). Figure 1f exhibits the morphology of POMOF**2**, exhibiting a uniform hexagonal prism shape with a length of about 2.5 μm, a width of about 600 nm, and a smooth surface. The results proved that nanoscale POMOF materials with regular morphologies were successfully obtained by stirring at room temperature. Moreover, the dye−removal performance is related to the charge properties of adsorbing materials; the Zeta potential of POMOF(**1**, **2**) powder was measured, demonstrating that POMOFs exhibited a negative potential in aqueous solutions, as shown in Table S1. The obtained POMOF(**1**, **2**) materials were used to conduct dye adsorption experiments.

### 2.2. Dye-Adsorption Performance of POMOF(**1**, **2**) Nanomaterials

**Figure 2:**
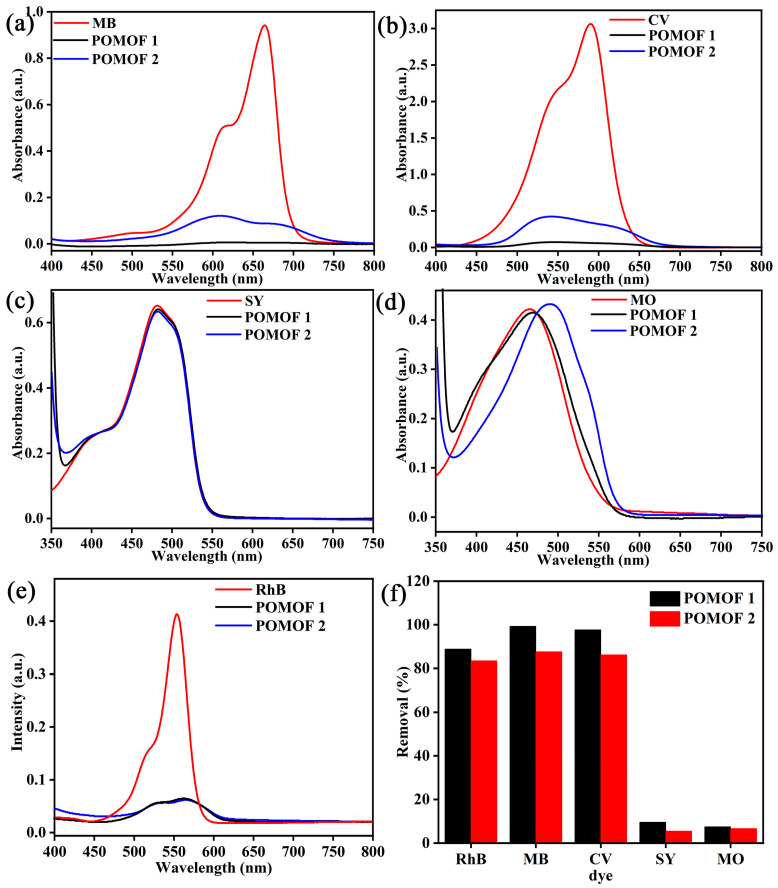
The UV−vis adsorption spectra of dye solutions: (**a**) MB, (**b**) CV, (**c**) SY, (**d**) MO, and (**e**) RhB before and after adsorbed by POMOF (**1**, **2**); (**f**) Removal rates of POMOF (**1**, **2**) for different dyes. (5 mg of POMOF(**1**, **2**), 12 mg/L and 10 mL of dye solutions, adsorption time 30 min).

Organic dye pollution in wastewater has received widespread attention. The removal efficacy of POMOFs concerning organic dyes was investigated based on their stabilized framework structure [28]. Five different dyes (three cationic: CV, RhB, and MB; two anionic: MO and SY) were selected for the adsorption experiments, and their structures are depicted in Figure S3. The standard curves of RhB, MB, and CV concentrations are shown in Figure S4. The two types of POMOF (**1**, **2**) materials were used as adsorbents. The initial dye conditions were 10 mL at 12 mg/L, with an adsorbent dosage of 5 mg, and the dye adsorption experiments were analyzed using UV−vis spectrophotometry. Figure 2 illustrates the adsorption effects of the two samples on these organic dyes. It was evident that the adsorption efficiency for all cationic dyes surpassed 90% in 30 min; in particular, the adsorption of MB and CV for POMOF**1** reached 100% and 99%, respectively (Figure 2a,b,e), while minimal adsorption was observed for anionic dyes (Figure 2c,d). It can be observed that the removal efficiency of POMOF**1** for cationic dyes was about 10% better than that of POMOF**2** (Figure 2f). This result may be attributed to the larger surface area of the porous rhombic dodecahedron of POMOF**1** compared to the smooth hexagonal prism structure of POMOF**2**, in addition to the more negative Zeta potential of POMOF**1** (Table S1).

However, despite the excellent filtration properties of the POMOFs, the inherent characteristics of nanoparticles could pose challenges to their separation from liquid environments. In order to increase recyclability and simplify post−processing, a carrier capable of holding powder materials is required [30]. In this study, polyacrylonitrile was chosen as a suitable substrate to combine with POMOF**1**, forming POMOF**1**/PAN NFM. The PAN exhibited durability and high thermal resistance, as well as low cost, which are crucial for ensuring its minimal performance impact on POMOF**1** nanoparticles [31].

### 2.3. Characterization of POMOF**1**/PAN NFM

**Figure 3:**
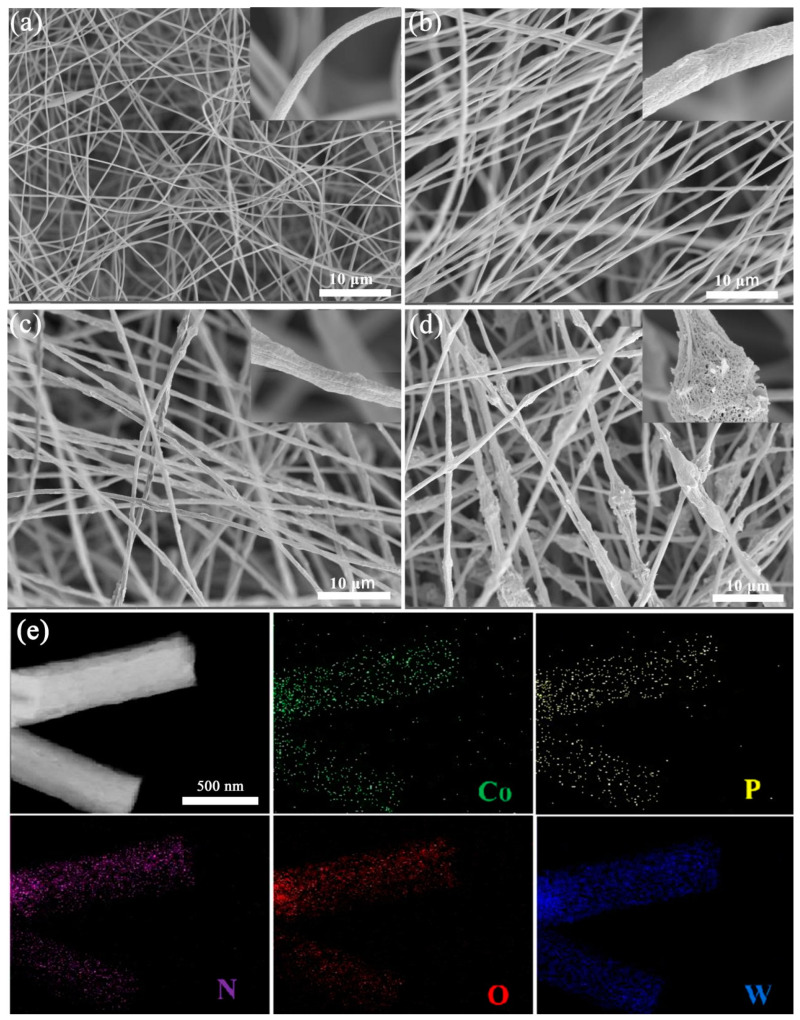
SEM images of POMOF**1**/PAN composite nanofibers with different POMOF**1** contents: (**a**) 0 wt%, (**b**) 3 wt%, (**c**) 5 wt%, and (**d**) 10 wt%; (**e**) EDS mapping of 5% POMOF**1**/PAN NFM: Co, P, N, O, and W elements.

The nanofiber membranes were prepared using different proportions of POMOF**1**: 0 wt%, 3 wt%, 5 wt%, and 10 wt% to yield PAN and POMOF**1**/PAN NFM, respectively. The prepared nanofiber membranes underwent fundamental characterization and performance testing. The microstructure and morphology of the membranes were investigated using SEM. As shown in Figure 3a, the morphology of a typical PAN NFM revealed a film comprising fibers with uniform rough surfaces. These fibers exhibited random and staggered orientations, with an average diameter of approximately 450 nm. At low power magnification (Figure 3b), the structure of 3 wt% POMOF**1**/PAN NFM was similar to that of PAN NFM, with the exception that the diameter of the nanofibers became thicker: it was about 1.2 μm. As the concentration of POMOF**1** increased (from 3 wt% to 5 wt%), the diameter of the fibers became uneven and POMOF**1** was observed in the nanofibers, as some nanoparticles started to locally accumulate on the fiber’s surface (Figure 3c). However, compared to other nanofiber membranes with different proportions, the 5 wt% fiber maintained the highest porosity and specific surface area, with the maximum specific surface area and pore size reaching 24.38 m^2^/g and 43.00 nm, respectively (Table S2). The notable porosity and specific surface area facilitated the diffusion of dye solutions within the POMOF**1**/PAN NFM, thereby facilitating rapid dye adsorption. When the POMOF**1** content reached 10 wt%, nanoparticles started to substantially accumulate in the nanofiber and the diameter of the fibers increased (Figure 3d). This increase in diameter could be attributed to an excessive quantity of POMOF**1**, which resulted in sample aggregation and irregular fiber formation. This, in turn, caused clogging, thus reducing efficiency due to excess nanoparticles [32]. The Energy−dispersive X−ray spectroscopy (EDS) mapping images in Figure 3e indicate uniform distributions of Co, P, N, O, and W elements across the 3 wt% POMOF**1**/PAN NFM material, thus confirming the successful synthesis of POMOF**1**/PAN nanofiber membranes via electrostatic spinning.

**Figure 4:**
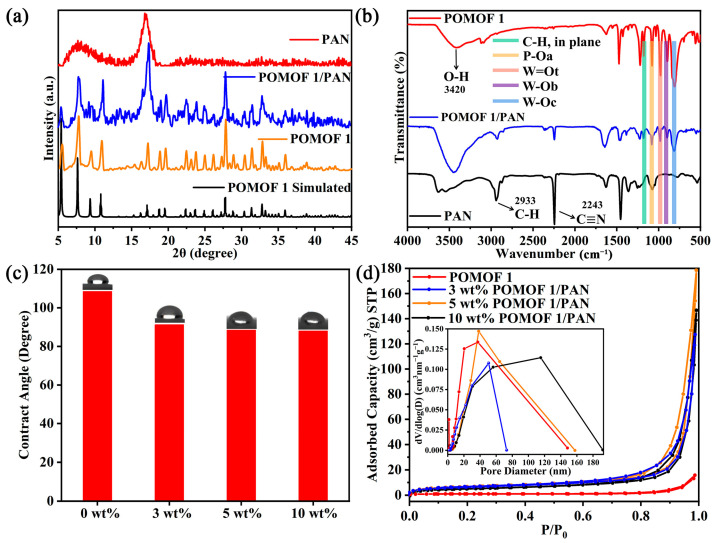
(**a**) The XRD patterns and (**b**) FT−IR spectra of PAN NFM, POMOF**1** powder, and POMOF**1**/PAN NFM; (**c**) water contact angle and (**d**) BET for different contents of POMOF**1** membranes.

The composition of the membranes was assessed using XRD and IR. Figure 4a displays the corresponding XRD patterns of the PAN NFM and POMOF**1**/PAN NFM. The XRD spectrum of the POMOF**1** powder was in good agreement with the data reported previously [30], indicating the successful preparation of POMOF**1** nanomaterials with excellent crystallinity. The characteristic diffraction peak at 16.8° in the XRD pattern of PAN NFM was attributed to the crystal planes (100) of PAN [33]. The XRD pattern of POMOF**1**/PAN NFM revealed characteristic peaks from both PAN and POMOF**1**, signifying that the original crystallinity of POMOF**1** was preserved during the spinning process, and it was successfully integrated with PAN NFM. The FT−IR images of the PAN NFM, the POMOF**1** powder, and POMOF**1**/PAN NFM are shown in Figure 4b. PAN NFM exhibited characteristic absorption peaks at 2243 cm^−1^ and 2933 cm^−1^, corresponding to the stretching vibration of C≡N and the bending vibration of C-H, respectively. The asymmetric tensile vibrations of POMOF**1** at 806 cm^−1^, 879 cm^−1^, 956 cm^−1^, and 1061 cm^−1^ correspond to the bonds of (W−Oc), (W−Ob), (W=Ot), and (P−Oa) in PW_12_, and the C−H characteristic vibrations of the dpdo ligand at 1180 cm^−1^. The infrared functional group at 3420 cm^−1^ represented O−H stretching vibrations [28,34]. In the POMOF**1**/PAN NFM, all characteristic absorption peaks of both PAN and POMOF**1** were distinctly observed, providing further confirmation of the successful combination of POMOF**1** nanoparticles with PAN NFM.

The thermal stability of PAN NFM and POMOF**1**/PAN NFM were assessed through thermogravimetric analysis (TGA) in N_2_ atmosphere, further demonstrating the successful preparation of composite materials, as shown in Figure S5. In the TGA spectra of the PAN NFM, a loss of approximately 2% was observed at around 100 °C; this was likely due to residual solvents and water molecules. A significant loss occurred at around 270 °C (approximately 40%), indicating polymer decomposition. Subsequently, a more moderate decomposition trend followed, and this was possibly associated with the dehydrogenation reaction and the ultimate decomposition of PAN NFM, resulting in a residual amount of 44.3% at 750 °C [35]. The POMOF**1**/PAN NFM exhibited a mere weight loss of 20% at approximately 270 °C while retaining a residual amount of 55.49% at 750 °C. This higher thermal stability in comparison to the PAN NFM can be attributed to the presence of POMOF**1** in POMOF**1**/PAN NFM. The addition of POMOF**1** significantly improved the stability of the composite. The results indirectly proved the successful preparation of POMOF**1**/PAN NFM.

The surface wettability of POMOF**1**/PAN membranes was assessed using a water contact angle (WCA) comparison test. A lower water contact angle indicates superior wettability, facilitating the permeation of organic wastewater and enhancing filtration separation efficiency [36]. In Figure 4c, PAN NFM exhibited a water contact angle of 108.49°, which could be attributed to its internal porosity and smooth surface, resulting in a substantial contact angle. Conversely, the hydrophilicity of POMOF**1**/PAN NFM was enhanced by incorporating POMOF**1** nanoparticles, resulting in a contact angle of approximately 90°. The specific surface area, pore volume, and pore nature of the composite membranes are important factors for pollutant removal and can be determined through N_2_ adsorption analysis using the Brunauer–Emmett–Teller (BET) method [37]. Figure 4d illustrates the N_2_ adsorption–desorption isotherms of the POMOF**1** and POMOF**1**/PAN NFM. The N_2_ adsorption–desorption isotherms displayed a characteristic type IV isotherm with a hysteresis loop, which was due to the porous structure of PAN nanofiber membranes. A summary of the Brunauer–Emmett–Teller (BET) specific surface area, average pore size, and pore volumes of POMOF**1** and POMOF**1**/PAN NFM are provided in Table S2. The 5 wt% POMOF**1**/PAN NFM (24.38 m^2^/g, 0.26 cm^3^/g) exhibited the highest surface area and pore volume compared with POMOF**1** (1.78 m^2^/g, 0.023 cm^3^/g), 3 wt% POMOF**1**/PAN NFM (22.09 m^2^/g, 0.18 cm^3^/g), and 10 wt% POMOF**1**/PAN NFM (20.85 m^2^/g, 0.22 cm^3^/g). The average pore size of POMOF**1**/PAN NFM calculated with the Barrett–Joyner–Halenda (BJH) method slightly changed within the range from 33.64 nm to 43.00 nm [38]. The high porosity and excellent hydrophilicity of the membrane enhanced liquid transport, ultimately improving filtration performance.

### 2.4. Dye Separation Properties of POMOF**1**/PAN NFM

#### 2.4.1. Adsorption and Separation of Cationic and Anionic Dyes

**Figure 5:**
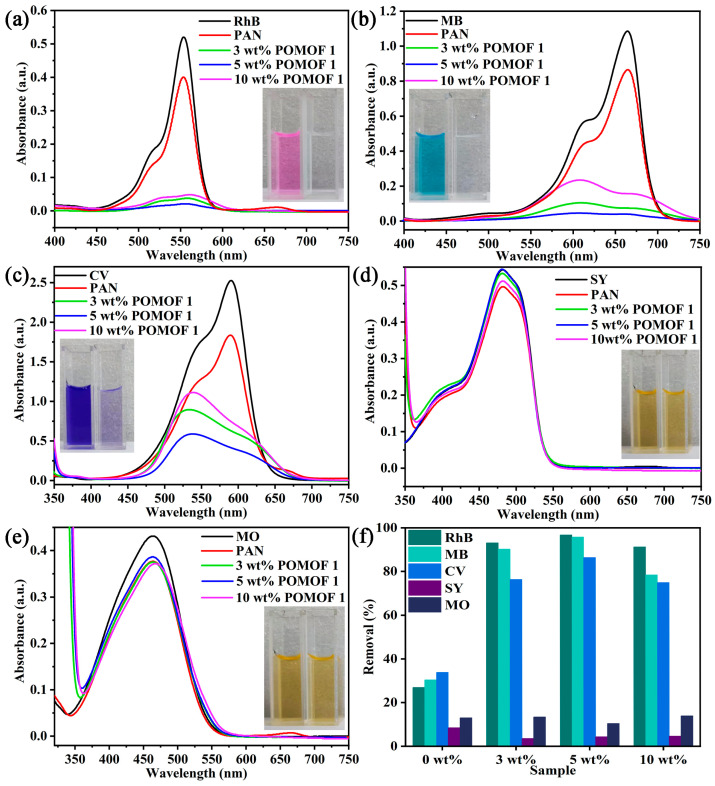
Adsorption properties of various membranes for different types of dyes: the UV–vis adsorption spectra of dyes (**a**) RhB, (**b**) MB, (**c**) CV, (**d**) SY, and (**e**) MO before and after filtration using different ratios of POMOF**1**/PAN NFM; (**f**) the corresponding removal efficiency. (Insets: photographs of the dye solutions with color changing) (The tests were performed with an initial dye condition of 10 mL at 12 mg/L, using a fixed–size (3.14 cm^2^) filter membrane).

In order to evaluate the filtration and separation ability of the doped POMOF**1** composite membranes, three kinds of cationic dyes (RhB, MB, and CV) and two anionic dyes (SY and MO) were used as diagnostic reagents to explore the dye removal performance of the membranes, as shown in Figure 5. The tests were performed with an initial dye condition of 10 mL at 12 mg/L using a fixed–size (3.14 cm^2^) filter membrane. The filtrate was used to calculate the dye removal ratio capacity. The removal efficiencies of three cationic dyes (RhB, CV, and MB) by doped composite membranes with varying proportions of POMOF**1** are illustrated in Figure 5a–c. The pure PAN NFM also displayed some removal efficiency for the three cations dyes, with removal rates of 27.5% (RhB), 30.3% (CV), and 32.3% (MB), respectively, likely due to the presence of cyano in the PAN, which has an affinity for cationic dyes. Furthermore, the membranes with different concentrations of POMOF**1** had different removal rates for the dye, with 5 wt% POMOF**1**/PAN NFM showing the best filtering effect for the three cationic dyes (RhB, MB, and CV). The filtration efficiencies of the membranes for RhB dye were 27.5% (PAN), 93.1% (3 wt% POMOF**1**), 96.7% (5 wt% POMOF**1**), and 91.2% (10 wt% POMOF**1**), respectively. The illustrations depicted the color change of the organic dyes before and after adsorption, showing that the solutions in the container were colorless and transparent after filtering, with the exception of CV. POMOF**1**/PAN NFM exhibited definite filtration and separation capabilities for cationic dyes, and this can be attributed to its distinct chemical structure, which featured a substantial number of negative charges on its surface [39,40]. In addition, with respect to anionic dyes, it was observed that the prepared membranes had almost no filtration effects on SY and MO, and this can be visually observed from the photos of the dye solution before and after filtration, as shown in Figure 5d,e. Figure 5f illustrates the removal rates of POMOF**1**/PAN NFM with different POMOF**1** contents for various organic dyes. The effect of 5 wt% POMOF**1**/PAN NFM was the most significant, achieving filtration separation rates of 96.7%, 95.8%, and 86.4% for cationic dyes RhB, MB, and CV, respectively. However, for the anionic dyes, MO and SY, the removal rates were only 10.4% and 6.4%, respectively. This indicated that POMOF**1** played a major role in cationic filtration processes, while PAN played a secondary role in the filtration processes. Figure S6 illustrates the color alterations of the membrane’s surface pre– and post–filtration. Following separation, a noticeable color change occurred on the surface of POMOF**1**/PAN NFM due to the adsorption of the cationic dye.

**Figure 6:**
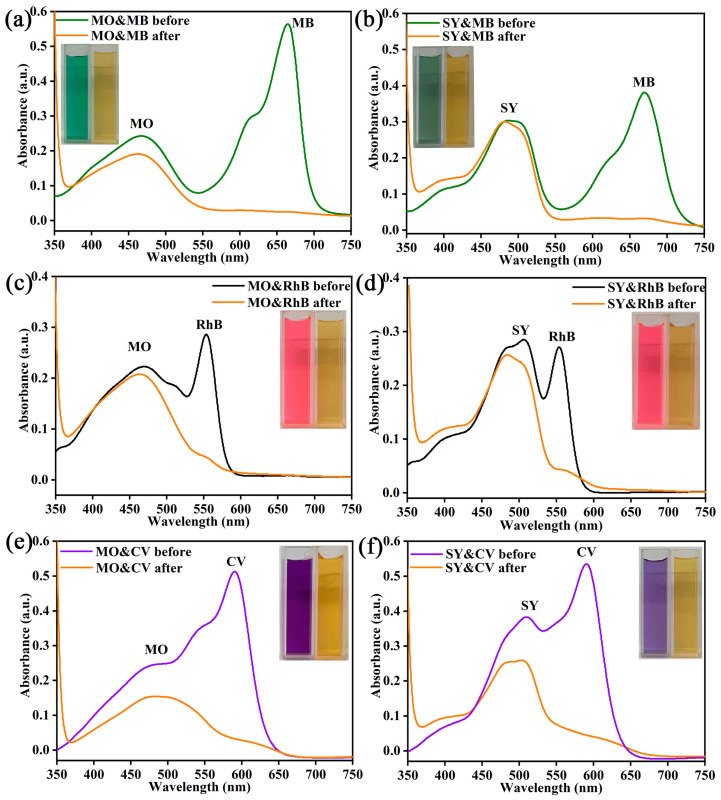
The UV–vis spectra and corresponding pictures of mixed dyes (10 mL) before and after filtration by 5 wt% POMOF**1**/PAN NFM: (**a**) MO and MB, (**b**) SY and MB, (**c**) MO and RhB, (**d**) SY and RhB, (**e**) MO and CV, and (**f**) SY and CV (insets: photographs of the dye solutions with color changes).

Considering the differences in the adsorption of monocomponent anionic and cationic dyes by the membranes, the selective separation ability of 5 wt% POMOF**1**/PAN NFM for mixed dyes solution was explored at room temperature by filtering six mixed dyes, combining anionic dyes (SY, MO) with cationic dyes (CV, RhB, and MB). The experiments were conducted using 10 mL of the mixed dyes, and all experiments were carried out under the same conditions. The type and concentration of residual dye were determined by recording the UV–vis absorption spectrum of the filtered solution, as shown in Figure 6. Employing the cationic dye MB and the anionic dye MO as examples, the UV–vis spectra and physical pictures are exhibited in Figure 6a. The absorbance of MB (at 664 nm) in the filtered solution decreased to 0 and the removal rate was calculated to be 100%. However, the removal rate of the anionic dye MO in the mixed solution was less than 18%, denoting that the MB was selectively adsorbed. Furthermore, the color of the mixture MO and MB (green) completely changed to the color of the anionic dye (yellow) after filtration, indicating the selective adsorption capacity of POMOF**1**/PAN NFM relative to cationic MB (inset of Figure 6a). The UV–vis absorption spectra of SY and MB, MO and RhB, SY and RhB, SY and CV, and MO and CV mixed dyes before and after filtration were measured. As shown in Figure 6b–d, 5 wt% POMOF**1**/PAN NFM also revealed similar performance for the other separation experiments. The removal rates were higher than 95% for cationic dyes and the color of the mixed solution changed to the color of a single anionic dye. Hence, POMOF**1**/PAN NFM can function as a selective filter for the segregation of organic dyes based on the opposite charges [41]. The selective absorption was ascribed to the electrostatic interactions between the POMOF**1** framework and cationic dyes. Conversely, in the case of anionic dyes, the negative charge on POMOF**1** can result in repulsion. Interestingly, for the mixed dyes MO and CV, and SY and CV, 5 wt% POMOF**1**/PAN NFM not only removed the cationic CV sufficiently but also exhibited a certain filtration effect on the anionic MO and SY dyes (Figure 6e,f).

#### 2.4.2. Adsorption Kinetics and Isotherms

**Figure 7:**
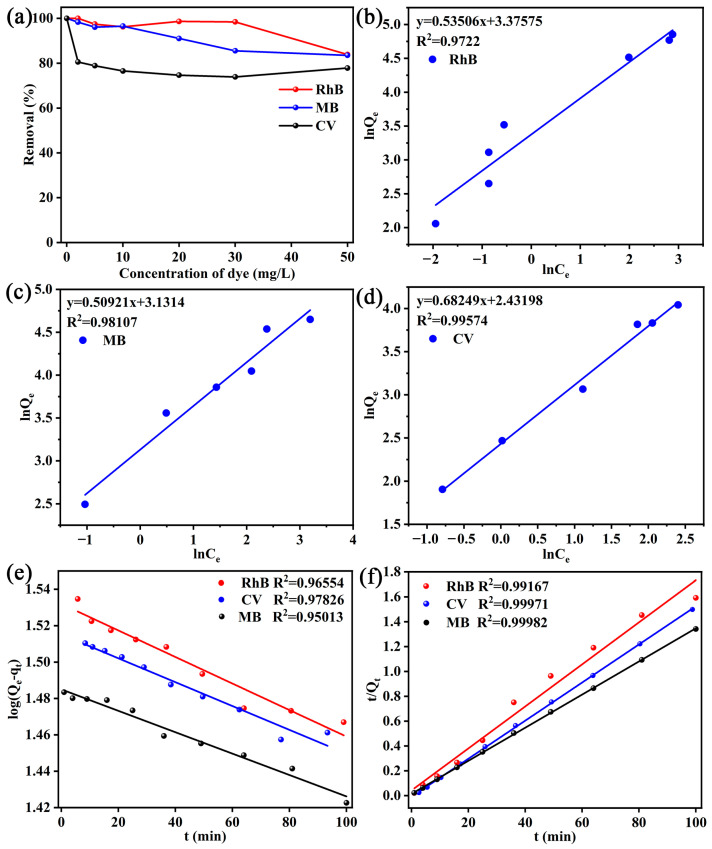
(**a**) The removal rates of different concentrations of dyes by POMOF**1**/PAN NFM filtration; (**b**−**d**) Freundlich isotherm models of RhB, MB, and CV; (**e**) the pseudo–first–order kinetic model and (**f**) the pseudo–second–order kinetic model.

The adsorption isotherm model was calculated by filtering different concentrations of dyes through POMOF**1**/PAN NFM and the nature of the dye–adsorbent interaction was studied. Figure 7a demonstrates the removal of three cationic dyes using 5 wt% POMOF**1**/PAN NFM at dye concentrations ranging from 2 to 50 mg/L. It was noted that when the concentration reached 50 mg/L, the removal rates of RhB, MB, and CV were 83.9%, 83.5%, and 77.8%, respectively, indicating a significant effect on the removal of cationic dyes. Using Formula (2) to process the absorption data for various concentrations of dyes, the saturated adsorption capacity of the film for three dyes could be determined. Figure S7 demonstrates that POMOF**1**/PAN NFM exhibited adsorption capacities of 180.9 mg/g (RhB), 127.4 mg/g (MB), and 64.2 mg/g (CV). The nature of the interaction between the dye and adsorbent was investigated using the adsorption isotherm model. Figure 7b,d and Figure S8 depict the Freundlich and Langmuir adsorption isotherm models for dye adsorption. The Freundlich thermodynamic coefficients (R^2^) of RhB, MB, and CV were 0.9722, 0.98107, and 0.99574, respectively, and the R^2^ values of the Langmuir model were 0.92429, 0.96561, and 0.92681. The higher R^2^ values for the three dyes obtained from the Freundlich model compared to the Langmuir model indicated a multilayer adsorption process [42,43]. The adsorption mechanism and rate control steps of dye adsorption on 5 wt% POMOF**1**/PAN NFM were investigated using pseudo–first–order and pseudo–second–order kinetic models. As shown in Figure S9, the adsorption capacity of the composite membrane increased rapidly during the first 3 min, after which the rise rate gradually stabilized. The increase in the adsorption efficiency of the dyes in the initial stage can be attributed to the presence of abundant active adsorption sites on the composite membrane. With time, the number of available active adsorption sites gradually decreases, resulting in a decrease in the interaction with the organic dye [44]. Figure 7e,f displays the fitted curves of the simulated kinetic models. The pseudo–first–order kinetic coefficients for RhB, MB, and CV are 0.96554, 0.95013, and 0.97826, respectively, and the second–order R^2^ values are 0.99167, 0.99982, and 0.99971. The results demonstrated a better fit relative to the pseudo–second–order kinetic model, with correlation coefficients (R^2^) exceeding 0.99, surpassing those obtained from the pseudo–first–order model [45]. This confirmed that the interactions between POMOF**1**/PAN NFM and RhB, MB, and CV were predominantly governed by chemical processes. Consequently, the mechanism of adsorption of organic dyes by the adsorbent likely involved chemical processes that were facilitated by electrostatic interactions between the dyes and the adsorbent.

Moreover, Figure S10 shows the efficiency of removal of organic dyes from water with different water qualities, including tap water, river water, and deionized water, by filtration with 5 wt% POMOF**1**/PAN NFM. It can be observed that the dye filtration effect was almost constant under these three different water conditions. Therefore, POMOF**1**/PAN NFM can filter organic dyes under various concentrations and different water quality conditions, indicating its substantial practical applicability [46]. Additionally, Table S3 lists some information regarding the removal rates of the three cationic dyes, which includes the utilization of membranes prepared using different polymers or composite membranes synthesized through doping different MOFs or inorganics with PAN as the substrate. Moreover, from the analysis of the removal rates of the three dyes using the materials in the table and the amount of adsorbed dyes, it can observed that POMOF**1**/PAN NFM presented superior results in terms of the amount of adsorbed dye or the removal rate when compared to the other materials in the table [47].

### 2.5. Mechanism Investigation

**Figure 8:**
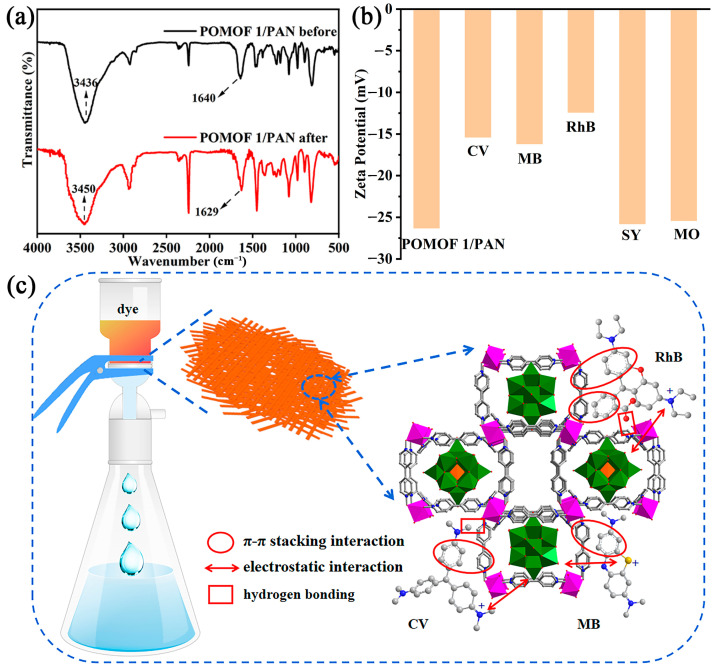
(**a**) The FT−IR spectra of 5 wt% POMOF**1**/PAN NFM before and after filtering RhB; (**b**) Zeta potential of 5 wt% POMOF**1**/PAN NFM soaking water and various dyes; (**c**) the adsorption mechanism of POMOF**1**/PAN NFM.

To further explain the excellent adsorption properties of POMOF**1**/PAN NFM, the adsorption mechanism of cationic dyes was established by analyzing the structure of the complexes, the FT–IR, and the Zeta potentials before and after dye adsorption. FT–IR spectroscopy was used to analyze the presence of characteristic functional groups in the materials, and the interaction between dye molecules and POMOF**1**/PAN NFM was explored according to the change in the vibration position of functional groups. Figure 8a shows the FT–IR spectra of 5 wt% POMOF**1**/PAN NFM before and after the adsorption of RhB, revealing a noticeable change in the vibration peak. Specifically, the peak corresponding to the stretching vibration of phenolic O–H and N–H in POMOF**1**/PAN NFM shifted from 3436 cm^−1^ to 3450 cm^−1^ after dye adsorption, suggesting the formation of hydrogen bonds between the membrane and RhB. The peak at 1640 cm^−1^ belongs to the stretching and bending vibrations of the C=C bonds in the POMOF**1**/PAN NFM, which shifted to 1629 cm^−1^ after adsorption of the dye; this may be because both the composite membrane and the cationic dye are rich in benzene rings, resulting in the π–π interactions between them [48,49]. As shown in Figure 8b and Table S1, the Zeta value of 5 wt% POMOF**1**/PAN NFM in deionized water was −26.5 mV, whereas the Zeta values of POMOF**1**/PAN NFM in the dye solution were −15.4 mV (RhB), −16.2 mV (MB), and −12.4 mV (CV), respectively. The Zeta potential analysis revealed a decrease in electronegativity after filtration, whereas the electronegativity of the two anionic dyes (MO, SY) electronegativity remained relatively unchanged (Table S1). In addition, the adsorption mechanism was dominated by electrostatic interactions between the negatively charged POMOF**1**/PAN NFM and the cationic dye (RhB, MB, CV) molecules, which is attributed to the abundant presence of negatively charged phenolic O–H groups in the POMOF**1**/PAN NFM. The structure of POMOF**1** is a 3D coordination polymer [Co_4_(dpdo)_12_]_∞_ framework, and the 3- charged heteropolyacid anion PW_12_ occupied three-quarters of the cavities in the framework [28]. From a structural point of view, the presence of heteropolyanions contributes to the electrostatic interaction between the filter membrane and the cationic dye. Therefore, electrostatic adsorption occurred between the organic dyes and POMOF**1**/PAN NFM. As shown in Figure 8c, the excellent adsorption capabilities can be attributed to hydrogen bonding, electrostatic adsorption, and π–π interaction.

**Figure 9:**
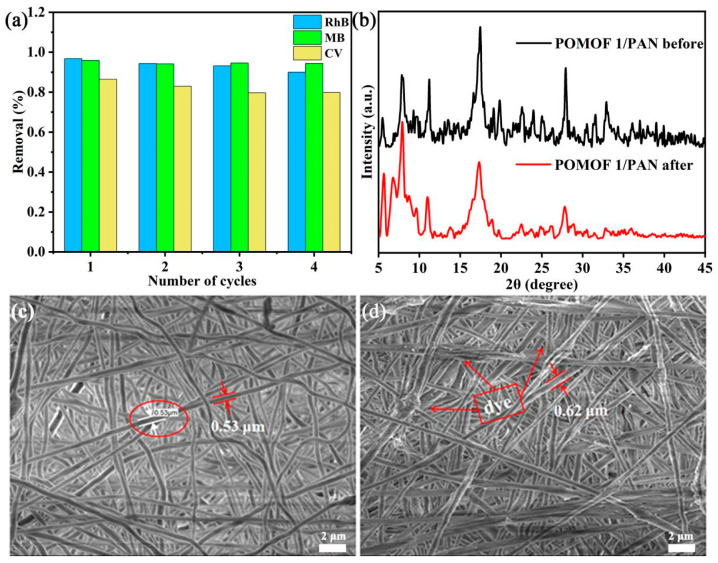
(**a**) The cyclic separation performance of 5 wt% POMOF**1**/PAN for RhB, MB, and CV; (**b**) XRD patterns of 5 wt% POMOF**1**/PAN before and after four cycles; SEM images (**c**) before and (**d**) after filtering dye.

Stability and reproducibility are crucial aspects of any adsorbent or filtration membrane used with green technology. To verify the above characteristics, the used 5 wt% POMOF**1**/PAN NFM was washed and dried using ethanol solution, following which it was subjected to the next cycle experiment. Figure 9a demonstrates that, after four filtration cycles, the removal rates of RhB, MB, and CV remained largely consistent, signifying the robust structural stability of POMOF**1**/PAN NFM during prolonged utilization. Figure 9b exhibits the XRD patterns of 5 wt% POMOF**1**/PAN NFM before and after filtration, revealing no discernible alterations and further suggesting that the preserved structure and crystallinity of the compounds were preserved [50]. As shown in Figure 9c,d, the SEM images of POMOF**1**/PAN NFM revealed the presence of adsorbed cationic dyes on the fiber’s surface, with no evident fiber breakage observed. Moreover, the diameter of POMOF**1**/PAN nanofibers was changed from 0.53 μm to 0.62 μm after RhB filtration. This suggests an interaction between POMOF**1**/PAN NFM and the cationic dye, while the NFM maintained its favorable fiber morphology. Thus, the XRD, FT−IR, and SEM spectra indicated minimal changes in crystallinity, crystal structure, and elemental composition, confirming the stability and durability of the sample. In addition, the leaching study of the 5 wt% POMOF**1**/PAN NFM was performed and tested by ICP−6000, the leaching amounts of Co and W were 1.8 and 2.2 ppm, respectively. In conclusion, POMOF**1**/PAN NFM demonstrates excellent recoverability and stability, ensuring prolonged service life, which is a crucial aspect for the efficient recovery and reuse of organic dyes in the context of wastewater treatment [51].

## 3. Materials and Methods

### 3.1. Reagents and Materials

Keggin-type heteropolyacid α-H_3_PW_12_O_40_·6H_2_O (PW_12_) and α-H_3_PMo_12_O_40_·14H_2_O (PMo_12_) were synthesized following the methods reported in the literature [52]. The 4,4′-bipyridine-N,N′-dioxide (dpdo) ligand was synthesized according to the method reported in [53]. Cobalt chloride hexahydrate (CoCl_2_·6H_2_O), polyacrylonitrile (PAN) (Mw = 150,000), acetic acid 36% aqueous solution, methylene blue (MB), rhodamine B (RhB), crystal violet (CV), methyl orange (MO), sunset yellow (SY), hydrogen peroxide, acetone, and acetonitrile were also used. All of the chemicals were of analytical purity grade and were purchased from commercial sources. The reagents were prepared using deionized water.

### 3.2. Synthesis of POMOF Nanomaterials

The POMOF**1** [Co_4_(dpdo)_12_][H(H_2_O)_21_(CH_3_CN)_12_][PW_12_O_40_]_3_ (POMOF−PW_12_) nanomaterial was synthesized using a modified version of the method reported in [28]. CoCl_2_·6H_2_O (35.5 mg, 0.15 mmol) and PW_12_ (450 mg, 0.15 mmol) were dissolved in water (5 mL) and heated at 80 °C for 4 h. Then, a 50 mL solution of acetonitrile/water (3:1, *v*/*v*) was combined with the previous solution. Subsequently, 110 mg (0.5 mmol) of dpdo dissolved in 10 mL of deionized water was added dropwise into the above mixture and magnetically stirred for 12 h at 25 °C; the mixture then stood for 6 h. The brick red powder was washed three times using acetonitrile and dried in a vacuum drying oven at 80 °C for 24 h (yield: 64.4%).

The POMOF**2** (POMOF−PMo_12_) nanomaterial was synthesized using the same method, except PW_12_ (450 mg, 0.15 mmol) was replaced by PMo_12_ (312 mg, 0.15 mmol) (yield: 55.4%).

### 3.3. Preparation of POMOF**1**/PAN Nanofiber Membrane (NFM)

POMOF**1**/PAN NFM was prepared using the electrospinning method. Firstly, PAN (500 mg) was vigorously stirred in 4 mL of DMF for 12 h. Then, a certain amount of POMOF**1** was dispersed in 0.5 mL acetonitrile and 1 mL DMF solution at the following proportions (0 wt%, 3 wt%, 5 wt%, and 10 wt%), and stirred for 20 min. The above two solutions were mixed uniformly while stirring and then transferred into a 10 mL plastic syringe connected with a 260 mm stainless-steel needle (0.5 mm inside diameter). Throughout the electrostatic spinning process, a positive voltage of 18 kV and a speed of 0.08 mL/h were used, along with a controlled temperature of 25 ± 2 °C and humidity of 60 ± 3%. A tin foil paper was placed on a grounding plastic hardboard 11 cm away from the needle and used to collect POMOF**1**/PAN composite nanofibers. After electrostatic spinning, the composite nanofiber membrane was dried in a vacuum drying oven at 60 °C for 12 h. Based on the amount of added POMOF**1**, the obtained composite nanofiber membranes were recorded as 3 wt%, 5 wt%, and 10 wt% POMOF**1**/PAN, respectively.

### 3.4. Dye Adsorption and Separation

Adsorption experiment for POMOF(**1**, **2**) powder materials: 5 mg POMOF powder material was uniformly dispersed in 10 mL of a single dye solution (12 mg/L) and stood for 30 min at room temperature. Then, the suspended mixture was centrifuged and the absorption spectrum of the supernatant was tested (350–800 mm).

Filtration separation experiments: the performance of POMOF**1**/PAN NFM was assessed by filtering aqueous solutions containing MB, RhB, and CV. The membrane was cut into a circular shape with a 2 cm diameter, providing an effective area of 3.14 cm^2^, and securely affixed onto the filter device. The concentration of filtered organic dyes was measured using a UV–vis spectrophotometer. The equations for the removal rate and adsorption capacity are as follows [54]:

(1) $$Remove=\frac{(C_{0}-C_{e})\cdot 100\%}{C_{0}}$$

(2) $$q_{e}=\frac{{(C}_{0}-C_{e})\cdot V}{m}$$

where C_0_ (mg·L^−1^) is the initial concentration of organic dyes, C_e_ (mg·L^−1^) is the concentration of organic dyes after filtration, V (mL) is the volume of filtration solution, m (mg) is the mass of the membrane at filtration, and q_e_ is the adsorbed amount of organic dye after filtration. To prepare the mixed dyes, a mixture of 12 mg/L MO and SY was combined with an equal volume of 12 mg/L RhB and MB, respectively. Additionally, 6 mg/L CV was mixed with an equal volume of 12 mg/L MO and SY to create the mixed dyes. Subsequently, 10 mL samples of each mixed dye were utilized for the selective dye separation test. Filtration was conducted using a filter unit housing with a fiber membrane positioned in the middle, facilitating the complete gravity–based filtration of 10 mL of dye within 90 s without applying pressure.

### 3.5. Adsorption Kinetics

In order to study the relationship between adsorption rate and the concentration of adsorbent and adsorbate during the adsorption process, the following tests were conducted: 10 mg of POMOF**1**/PAN NFM was immersed in a 100 mL aqueous solution containing 75 mg/L of cationic dyes (RhB, CV, and MB), and the adsorption process was carried out in a water bath shaker. Subsequently, 5 mL of the suspension was periodically taken and the UV–vis absorption spectrum of the supernatant was tested after centrifugation. Its dynamic model formula is as follows [45]:

The pseudo–first–order kinetic model (explaining the adsorption rate of adsorbents capturing solutes):

(3) $$log(Q_{e}-q_{t})=logQ_{e}-k_{1}t$$

The pseudo–second–order kinetic model (chemical reactions mainly dominate the adsorption process):

(4) $$\frac{t}{Q_{t}}=\frac{1}{k_{2}{Q_{e}}^{2}}+\frac{t}{Q_{e}}$$

where Q_e_ and q_t_ are the adsorption capacities (mg/g) at equilibrium and time t (min), respectively. Moreover, k_1_ and k_2_ represent the rate constants of the pseudo–first–order and pseudo–second–order kinetic models.

### 3.6. Adsorption Isotherm Determination

The equilibrium adsorption isotherms of various dyes with respect to POMOF**1**/PAN NFM were studied using Langmuir and Freundlich adsorption models to simulate their adsorption behavior [26].

The Langmuir model (homogeneous monolayer adsorption) is described as follows:

(5) $$\frac{C_{e}}{Q_{e}}=\frac{C_{e}}{Q_{max}}+\frac{1}{Q_{max}K_{L}}$$

The Freundlich model (multi–phase and multi–layer adsorption) is described as follows:

(6) $$lnQ_{e}=lnK_{F}+ln\frac{C_{e}}{n}$$

where Q_e_ is the equilibrium adsorption capacity (mg g^−1^), C_e_ is the equilibrium concentration (mg L^−1^), Q_max_ is the maximum adsorption capacity, K_L_ is Langmuir’s constant, K_F_ is Freundlich’s constant, and 1/n is the adsorption strength.

## 4. Conclusions

In this work, we successfully obtained two POMOF nanomaterials with porous rhomboid–shaped dodecahedral (POMOF–PW_12_) and hexagonal prism (POMOF–PMo_12_) structures using a simple green synthesis method. The POMOFs were found to possess good water stability and negative Zeta potential and can be employed for the adsorption of organic dyes from wastewater. Next, a POMOF/PAN nanofiber membrane was prepared using the electrospinning technique for the filtration of cationic dyes. The test results demonstrated that the 5 wt% POMOF**1**/PAN nanofiber membrane achieved the highest removal rates of 96.7%, 95.8%, and 86.4% for RhB, MB, and CV, respectively, which indicated that an appropriate amount of POMOF**1** helps to improve adsorption and the separation efficiency. Research using kinetic and isotherm models suggested that a multi–layer chemisorption–controlled process is the main adsorption approach. The separation mechanism involves π–π stacking, electrostatic adsorption, and hydrogen bond interactions. After four cycles, the filtration efficiency of the 5 wt% POMOF**1**/PAN NFM for cations dyes remained consistently above 90%, showcasing its strong stability and cycling capabilities. Hence, electrospinning polyoxometalate-based metal–organic frameworks filtration membranes facilitate the selective removal of target dyes from wastewater, showcasing their applicability in dye wastewater treatment. This study provides a practical and economical adsorption and separation material for cationic dyes, as well as constructive suggestions for the construction of new POMOFs materials with a large amount of charge for the treatment of substances in wastewater.

## Data Availability

Data are contained within the article and Supplementary Materials.

## Supplementary Materials

The following supporting information can be downloaded at: https://www.mdpi.com/article/10.3390/molecules29071493/s1, Figure S1: The structure of POMOF**1** (a) Structural unit diagram; (b) Coordination environment of cobalt center and dpdo ligand; (c) The 3D coordination polymers [Co_4_(dpdo)_12_]_∞_ framework along c axis; (d) The polyhedral diagram of the 3–charged heteropolyacid anion occupied three–quarters of the cavities, Figure S2: The XRD patterns of POMOF**1** at different pH conditions, Figure S3: The molecular structure of dyes, Figure S4: Standard curves of the dyes in aqueous solution (a) RhB, (b) MB and (c) CV, Figure S5: The TGA curves of POMOF**1** powder, POMOF**1**/PAN, and PAN NFM, Figure S6: Photos taken before and after membrane filtration, Figure S7: Saturation adsorption of RhB, MB, and CV for POMOF**1**/PAN NFM, Figure S8: Langmuir isotherm modeling of RhB, MB, and CV for POMOF**1**/PAN NFM, Figure S9: The adsorption effect of POMOF**1**/PAN NFM on MB and RhB over different time periods, Figure S10: Removal rate of cationic dyes in water of different qualities, Table S1: The Zeta potential of POMOF (**1**, **2**) powder and POMOF**1**/PAN NFM in different dye water solvents, Table S2: Specific surface area, pore size, and pore capacity of POMOF**1**/PAN NFM of different proportions, Table S3: Removal efficiency of RhB, MB, and CV by different materials. References [55,56,57,58,59,60,61,62,63,64,65,66,67,68] are cited in the Supplementary Materials.
