# Supplementary material for: Rapid, Massive, and Green Synthesis of Polyoxometalate-Based Metal–Organic Frameworks to Fabricate POMOF/PAN Nanofiber Membranes for Selective Filtration of Cationic Dyes
Source: Molecules. 2024 Mar 27;29(7):1493. doi: 10.3390/molecules29071493 (PMC11013096; doi:10.3390/molecules29071493)
Supplement: Supplementary file 1 [file molecules-29-01493-s001.zip › molecules-2866459-supplementary.pdf]

Supporting Information

Rapid, Massive, and Green Synthesis of Polyoxometalate-Based Metal–Organic Frameworks to Fabricate POMOF/PAN Nanofiber Membranes for Selective Filtration of Cationic Dyes

Jianping Li *,†, Zhaoke Yu †, Jiaming Zhang, Chengjie Liu, Qi Zhang, Hongfei Shi and Dai Wu *

College of Materials Science and Engineering, Jilin Institute of Chemical Technology, Jilin 132022, China; yu2576289101@163.com (Z.Y.); 13943133446@163.com (J.Z.); L18660052182@163.com (C.L.); 17696079502@163.com (Q.Z.); shihf813@nenu.edu.cn (H.S.)

* Correspondence: lijp156@nenu.edu.cn (J.L.); wudai@jlict.edu.cn (D.W.)

† These authors contributed equally to this work.

Content

S1. Apparatus and characterization.

S2. Supplementary Experimental.

S3. The structure of POMOF.

S4. XRD patterns of POMOF1 at different pH conditions.

S5. The Zeta potential of the materials.

S6. The molecular structure of dyes.

S7. The standard curves of the dyes.

S8. The specific surface area, pore size and pore capacity.

S9. The TGA curves of POMOF1 powder, POMOF1/PAN and PAN NFM.

S10. The photographs before and after membrane filtration.

S11. Removal rate of cationic dyes in different water quality.

S12. The effect of adsorption of dyes at different times by 5 wt% POMOF1/PAN NFM.

S13. Saturation adsorption and Langmuir isotherm modeling.

S14. Removal efficiency compared to the literature.

S1. Apparatus and characterization.

The morphology structure was characterized by scanning electron microscopy (SEM) (JSM-7610F Plus, Japan), and the samples were studied in the backscattered electron mode with an accelerating voltage of 5 kV and a distance of 8 mm, respectively. Fourier transform infrared spectroscopy (FT-IR) tested the analysis of the data before and after the separation of the membrane, in the range of 4000-400cm^{-1} (Bruker TENSORII, Germany). The concentration of the organic dye solution was tested with a ultraviolet-visible spectrophotometer (UV-vis) in the range of 800-200 cm^{-1} (Shimadzu UV-2600, Japan). Powder X-ray diffraction (XRD) was used to determine the crystal structure of the synthesized sample and the separated film (Dandong Haoyuan DX-2700BH, China). The N_2 adsorption-desorption isotherms was measured on BSD-PM2 (BeiShiDe instrument). The starting Angle was 5°-45°, the step width Angle was 0.02, the tube voltage was 40 V and the tube current was 30 A. The internal structure and possible elements in the fiber membrane and their distribution were analyzed using a transmission electron microscope (TEM) (JEOL, Japan Electronics, JEM-F200). Thermogravimetric analysis (TGA) was used to evaluate the thermal stability of the fiber membrane at a temperature range of 40-750 °C and a heating rate of 10 °C min^{-1} under nitrogen (50 mL·min^{-1}) atmosphere (TA corporation, Q50). The hydrophilicity of the membrane can be characterized by water contact Angle (WCA) (Shanghai Zhongchen digital technology Equipment Co., LTD, JC2000DM). Inductively coupled plasma (ICP-6000).

S2. Supplementary Experimental.

2.1 Synthesis of 4,4′-bipyridine-N,N′-dioxide (dpdo)

The dpdo ligand was synthesized according to a literature method [1]. First, a mixture of 4,4′-bipyridine (25.6 mmol, 4 g), acetic acid (25 mL), and 30% hydrogen peroxide (10 mL) was heated at 80° for 3 h. Then, the 7.5 mL of hydrogen peroxide was dropwise added to the above solution and maintained heating for 12 h. After the solution was cooled to room temperature and transferred to a beaker, a large amount of light yellow solid was precipitated during the 500 mL of acetone was added with stirring. The purified dpdo sample was separated by centrifugation and dried under vacuum at

60 °C for 24 h.

2.2 Hydrothermal synthesis of POMOF crystal

POMOF**1** ($\{[Co_4(dpdo)_{12}][H(H_2O)_{21}(CH_3CN)_{12}][PW_{12}O_{40}]_3\}_\infty$) crystals was prepared using the modified synthesis conditions based on the literature [2]. $CoCl_2·6H_2O$ (7.1 mg, 0.03 mmol) 、α-$H_3PW_{12}O_{40}·6H_2O$ (90 mg, 0.03 mmol) and dpdo (22 mg, 0.5 mmol) were dissolved in the mixed solution of acetonitrile (2 mL) and deionized water (4 mL) in turn. The mixture was transferred to a 25 mL teflon-lined stainless steel and heated at 80 °C for 24 h. After cooling to room temperature, reddish-brown block crystals was washed three times with acetonitrile and dried at room temperature.

POMOF**2** ($\{[Co_4(dpdo)_{12}][H(H_2O)_{21}(CH_3CN)_{12}][PMo_{12}O_{40}]_3\}_\infty$) crystals was prepared at the same method, except α-$H_3PW_{12}O_{40}·6H_2O$ (90 mg, 0.03 mmol) was replaced by α-$H_3PMo_{12}O_{40}·14H_2O$ (62.4 mg，0.03 mmol).

2.3 Zeta potential test

Typically, each sample powder (3 mg) was fully ground and then dispersed in deionized water (10 mL) by ultrasonication for 30 minutes. The zeta potential of each sample was measured three times by testing the suspension at the initial pH value.

2.4 Recyclability of POMOF1/PAN NFM

Four cycling experiments were performed to evaluate the reusability of POMOF1/PAN NFM. In each cycle, 10 mL (12 mg/L) of organic dye underwent filtration. Subsequently, the membrane was cleaned of organic dyes by ethanol rinsing before its next use, followed by drying at 60 °C in an oven for subsequent filtration and separation.

S3. The structure of POMOF1.

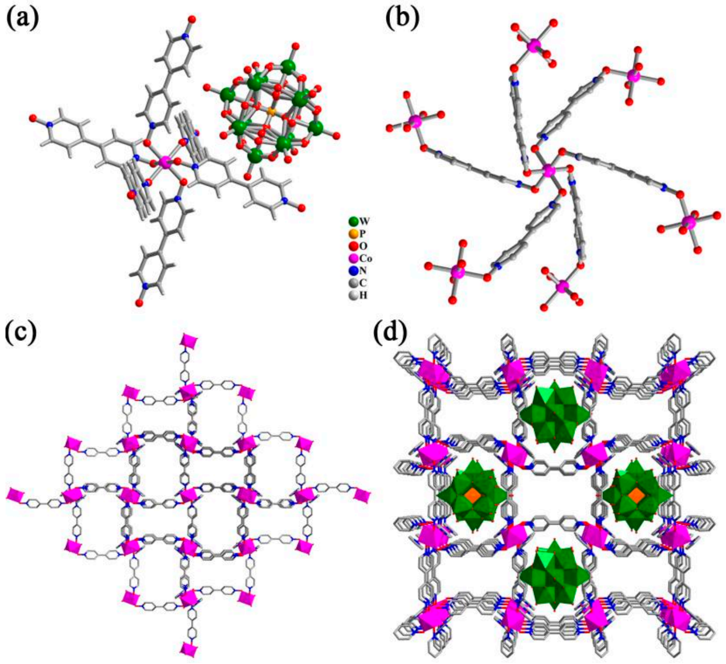

Figure S1. The structure of POMOF**1** (a) Structural unit diagram; (b) Coordination environment of cobalt center and dpdo ligand; (c) The 3D coordination polymers $[Co_4(dpdo)_{12}]_\infty$ framework along c axis; (d) The polyhedral diagram of the 3^- charged heteropolyacid anion occupied three-quarters of the cavities. (The possible water cluster that occupied a quarter of the cavities of the MOFs are omitted for clarity.) Co: purple, W: green, O: red, P: orange, N: blue, C: gray, H: white.

S4. XRD patterns of POMOF1 at different pH conditions.

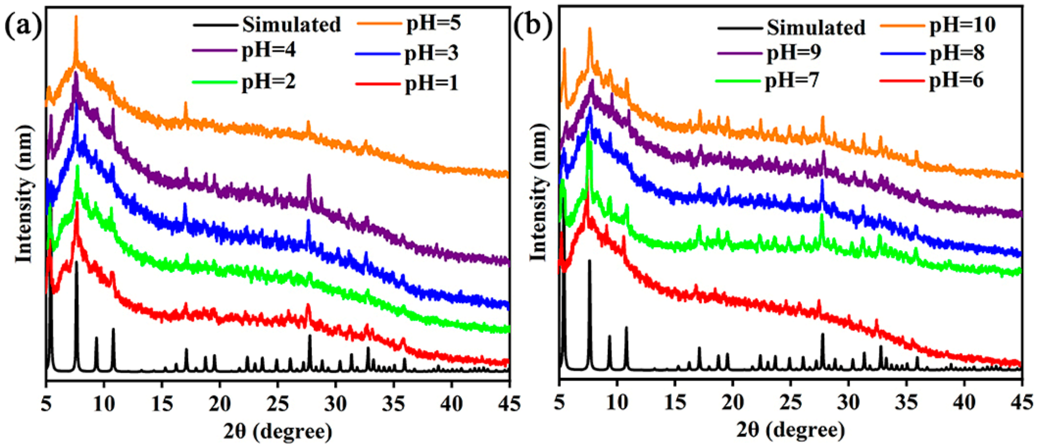

Figure S2. The XRD patterns of POMOF**1** at different pH conditions (Soak 30 mg POMOF**1** in 3 mL of solutions with different pH for 2 hours. The pH was adjusted with 0.1 M NaOH and 0.1 M HC1. After soaking, centrifuge and vacuum dry for the XRD test).

S5. The Zeta potential of the materials.

Table S1. The Zeta potential of POMOF (**1**, **2**) powder and 5wt% POMOF**1**/PAN NFM in different dye water solvents.

Sample	solvent	First (mV)	Second (mV)	Third (mV)	Average (mV)
POMOF**1**	H_2O	-32.6	-31.2	-32.3	-32.0
POMOF**2**	H_2O	-30.7	-31.1	-30.9	-30.9
POMOF**1**/PAN	H_2O	-26.3	-26.8	-26.5	-26.5
POMOF**1**/PAN	RhB (12 mg/L)	-14.9	-16.2	-15.2	-15.4
POMOF**1**/PAN	MB (12 mg/L)	-16.5	-15.6	-17.1	-16.2
POMOF**1**/PAN	CV (12 mg/L)	-12.9	-12.1	-12.3	-12.4
POMOF**1**/PAN	MO (12 mg/L)	-24.9	-25.9	-26.7	-25.8
POMOF**1**/PAN	SY (12 mg/L)	-24.9	-25.8	-25.6	-25.4

S6. The molecular structure of dyes.

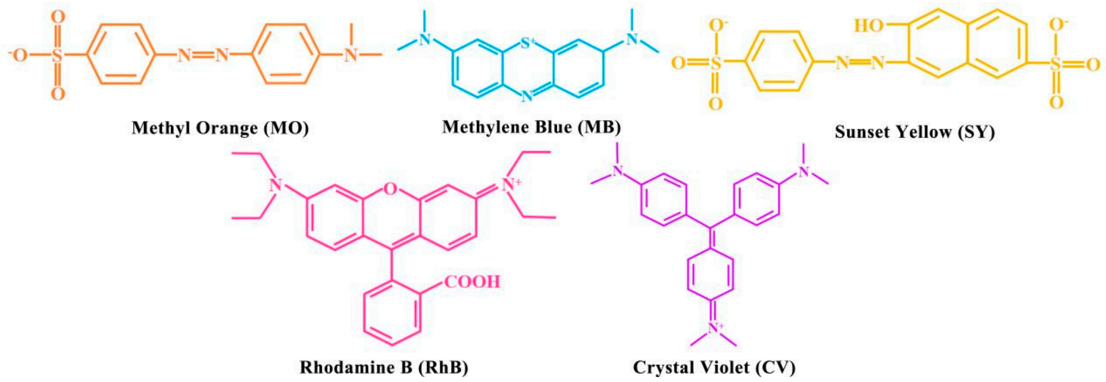

Figure S3. The molecular structure of dyes (MO and SY are anionic dyes; MB, RhB, and CV are cationic dyes).

S7. The standard curves of the dyes.

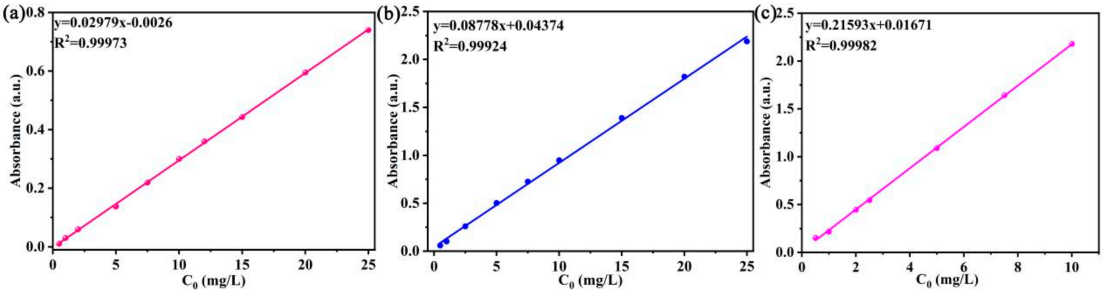

Figure S4. Standard curves of the dyes aqueous solution (a) RhB, (b) MB and (c) CV.

The standard curves of RhB and MB solutions were obtained at different concentrations of standard solutions (0.5-25 mg/L). CV standard curves were obtained in concentrations range of 0.5-10 mg/L.

S8. The specific surface area, pore size and pore capacity.

Table S2 Specific surface area, pore size and pore capacity of POMOF**1**/PAN NFM of different proportions.

Samples	Specific surface area (m^2/g)	Average pore size (nm)	Pore volume ($cm^3 \cdot g^{-1}$)
POMOF**1**	1.78	51.17	0.023
3wt% POMOF**1**/PAN	22.09	33.64	0.18
5wt% POMOF**1**/PAN	24.38	43.00	0.26
10wt% POMOF**1**/PAN	20.85	41.76	0.22

S9. The TGA curves of POMOF1 powder, POMOF1/PAN and PAN NFM.

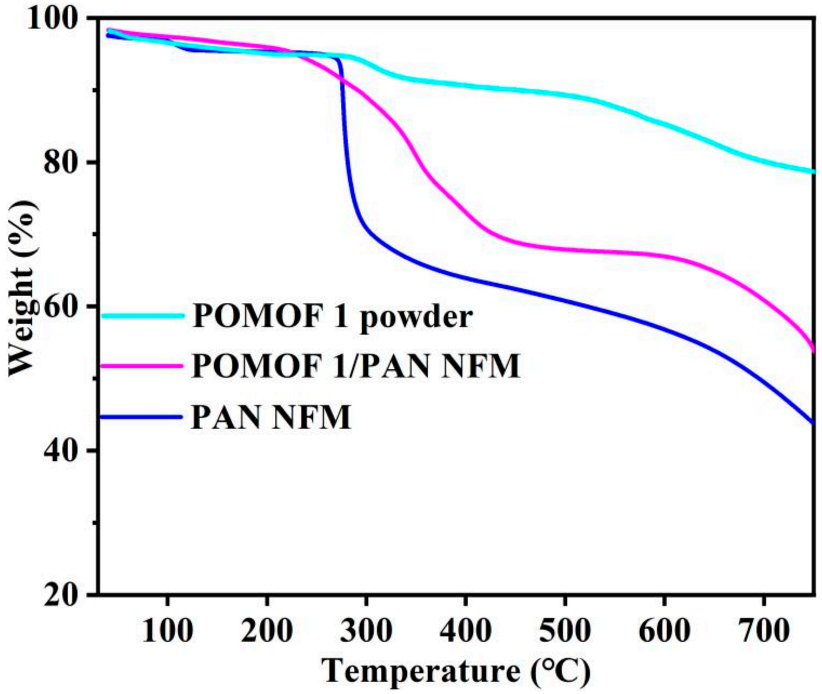

Figure S5. The TGA curves of POMOF**1** powder, POMOF**1**/PAN and PAN NFM.

S10. The photographs before and after membrane filtration.

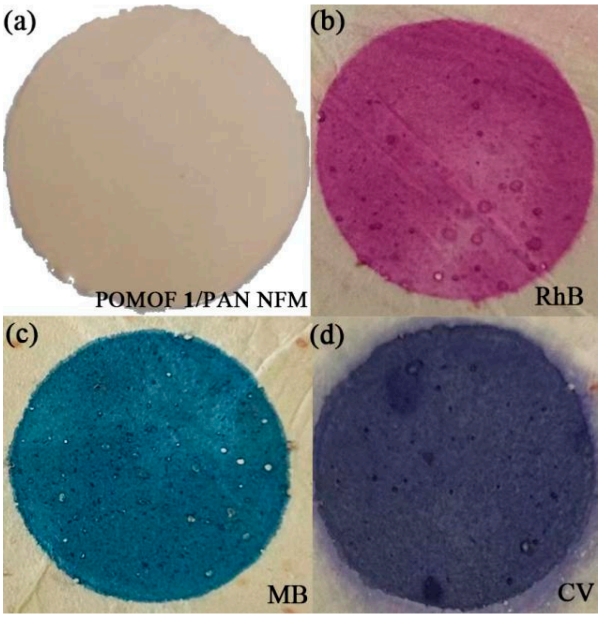

Figure S6. Photographs of POMOF**1**/PAN NFM before and after filtration different dyes (a) the fresh membrane; (b) RhB; (c) MB; (d) CV.

S11. Saturation adsorption and Langmuir isotherm modeling.

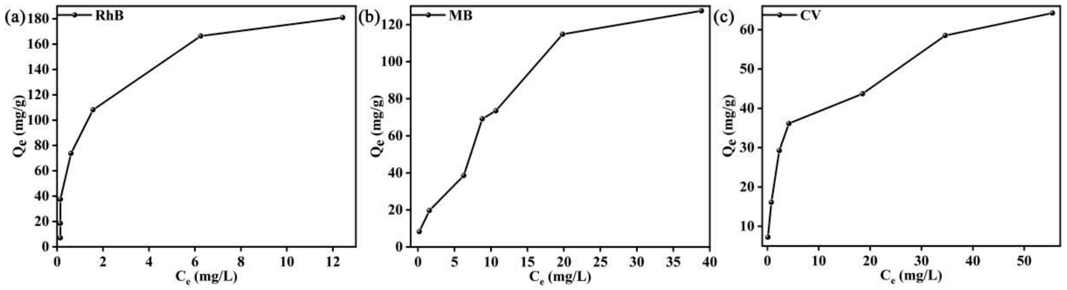

Figure S7. Saturation adsorption of RhB, MB and CV for POMOF**1**/PAN NFM.

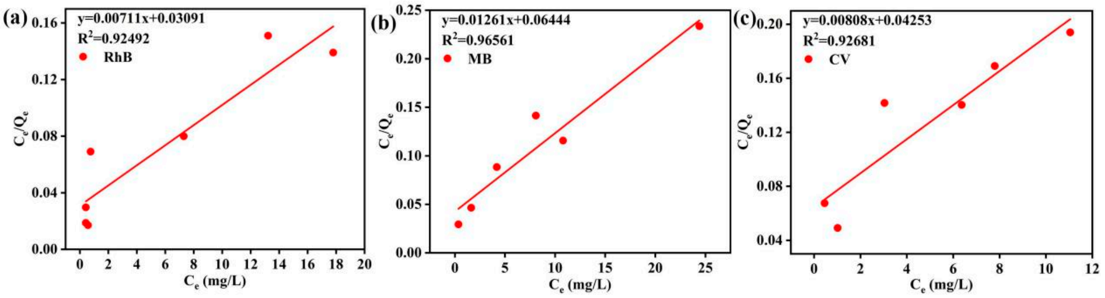

Figure S8. Langmuir isotherm modeling of RhB, MB, and CV for POMOF**1**/PAN NFM.

S12. The effect of adsorption of dyes at different times by 5 wt% POMOF1/PAN NFM

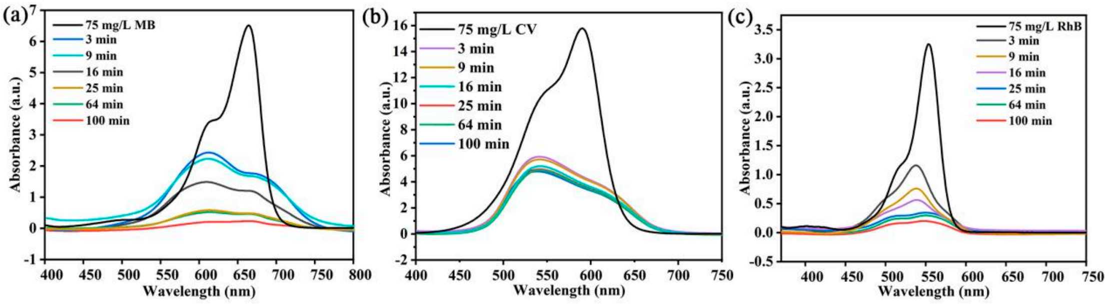

Figure S9. The UV-vis adsorption spectra of dyes solution (a) MB, (b) CV, (c) RhB in the different adsorption time by 5wt% POMOF**1**/PAN NFM.

S13. Removal rate of cationic dyes in different water quality.

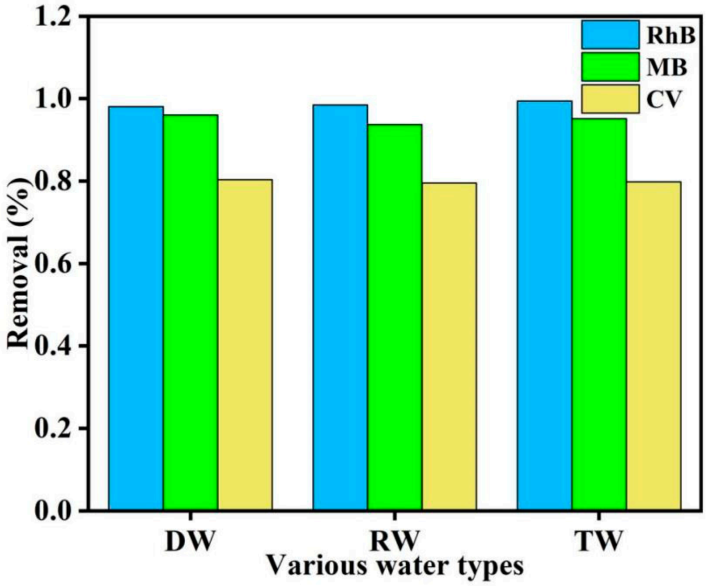

Figure S10. Removal rate of cationic dyes in different water quality (DW: Deionized water; RW: River water; TW: Tap water).

S14. Removal efficiency compared to the literature.

Table. S3 Removal efficiency of RhB, MB and CV by different materials.

Adsorbents	dye	Concentration	Qe	Dye removal rate	Ref.
ZIF-8/TOCN	RhB	10 mg/L	107 mg/g	93.8%	[3]
PAN-MIL-101(Fe)-NH_2	RhB	10 mg/L	333.33 mg/g	96.03%	[4]
pMIL-88A/PAN	RhB	20 mg/L	97.56 mg/g	94.4%	[5]
TSCB	MB	60 mg/L	58.9 mg/g	81%	[6]
bio-MOF-1@TPU	MB	20 mg/L	21.7 mg/g	94.04%	[7]
bio-MOF-1@PAN	MB	10 mg/L	20.68 mg/g	74.6%	[8]
LDH/GO/PVDF	MB	20 mg/L	120 mg/g	90%	[9]
PSF/MIL-101(Fe)-NH_2	RhB	10 mg/L	13.31 mg/g	95%	[10]
UiO-66/PGP	RhB	10 mg/L	31.3 mg/g	95.5%	[11]
CS/CD/MIL-68(Al)	MB	10 mg/L	60.61 mg/g	92.6%	[12]
CS/PVP/PVA@PEO	MB	30 mg/L	41.43 mg/g	83.91%	[13]
CS/PVP/PVA@PEO	CV	30 mg/L	30.97 mg/g	71.99%	[13]

GO/HEC/PGDE/Fe_3O_4	CV	50 mg/L	60.83 mg/g	73.65%	[14]
GO/HEC/PGDE/Fe_3O_4	MB	50 mg/L	69.40 mg/g	86.35%	[14]
TSCB	CV	60 mg/L	59.3 mg/g	96.35%	[6]
ZIF-101	CV	5 mg/L	7.86 mg/g	72%	[15]
ZIF-201	CV	5 mg/L	10.83 mg/g	76%	[15]
MSBA	CV	10 mg/L	49.1 mg/g	83.04%	[16]
Calixpyridinium-PMA	CV	10 mg/L	88.89 mg/g	81%	[17]
POMOF**1**/PAN NFM	RhB	12 mg/L	180.9 mg/g	96.7%	This work
POMOF**1**/PAN NFM	MB	12 mg/L	127.4 mg/g	95.8%	This work
POMOF**1**/PAN NFM	CV	12 mg/L	64.2 mg/g	86.4%	This work

References

1. Jia, J.; Blake, A.J.; Champness, N.R.; Hubberstey, P.; Wilson, C.; Schröder, M. Multi-Dimensional Transition-Metal Coordination Polymers of 4,4′-Bipyridine-*N*, *N*′-Dioxide: 1D Chains and 2D Sheets. *Inorganic Chemistry* **2008**, *47*, 8652-8664, doi:10.1021/ic800422g.
2. Duan, C.; Wei, M.; Guo, D.; He, C.; Meng, Q. Crystal Structures and Properties of Large Protonated Water Clusters Encapsulated by Metal-Organic Frameworks. *Journal. American. Chemistry. Society.* **2010**, *132*, 3321-3330, doi:10.1021/ja907023c.
3. Song, Y.; Seo, J.Y.; Kim, H.; Beak, K.Y. Structural Control of Cellulose Nanofibrous Composite Membrane with Metal Organic Framework (ZIF-8) for Highly Selective Removal of Cationic Dye. *Carbohydrate Polymers* **2019**, *222*, 115018, doi:10.1016/j.carbpol.2019.115018.
4. Jia, J.; Wu, H.; Xu, L.; Dong, F.; Jia, Y.; Liu, X. Removal of Acidic Organic Ionic Dyes from Water by Electrospinning a Polyacrylonitrile Composite MIL101(Fe)-

NH_2 Nanofiber Membrane. *Molecules* **2022**, *27*, 2035, doi:10.3390/molecules27062035.

5. Wu, H.; Xu, L.; Jia, J.; Dong, F.; Jia, Y.; Liu, X. In Situ Electrospun Porous MIL-88A/PAN Nanofibrous Membranes for Efficient Removal of Organic Dyes. *Molecules* **2023**, *28*, 760, doi:10.3390/molecules28020760.
6. Salah Omer, A.; A.El Naeem, G.; Abd-Elhamid, A.I.; O.M. Farahat, O.; A. El-Bardan, A.; M.A. Soliman, H.; Nayl, A.A. Adsorption of Crystal Violet and Methylene Blue Dyes Using a Cellulose-Based Adsorbent from Sugercane Bagasse: Characterization, Kinetic and Isotherm Studies. *Journal of Materials Research and Technology* **2022**, *19*, 3241–3254, doi:10.1016/j.jmrt.2022.06.045.
7. Jia, Z.; Li, T.; Wang, B.; Zhang, J.; Zhang, Z. A Robust Metal-Organic Framework Nanofibrous Membrane as Filter for Fast Separation of Cationic Dyes from Aqueous Solution. *Materials Letters* **2022**, *325*, 132908, doi:10.1016/j.matlet.2022.132908.
8. Li, T.; Liu, L.; Zhang, Z.; Han, Z. Preparation of Nanofibrous Metal-Organic Framework Filter for Rapid Adsorption and Selective Separation of Cationic Dye from Aqueous Solution. *Separation and Purification Technology* **2020**, *237*, 116360, doi:10.1016/j.seppur.2019.116360.
9. Zeng, H.; Yu, Z.; Peng, Y.; Zhu, L. Environmentally Friendly Electrostatically Driven Self-Assembled LDH/GO/PVDF Composite Membrane for Water Treatment. *Applied Clay Science* **2019**, *183*, 105322, doi:10.1016/j.clay.2019.105322.
10. Vinothkumar, K.; Shivanna Jyothi, M.; Lavanya, C.; Sakar, M.; Valiyaveettil, S.; Balakrishna, R.G. Strongly Co-Ordinated MOF-PSF Matrix for Selective Adsorption, Separation and Photodegradation of Dyes. *Chemical Engineering Journal* **2022**, *428*, 132561, doi:10.1016/j.cej.2021.132561.
11. Fang, S.Y.; Zhang, P.; Gong, J.L.; Tang, L.; Zeng, G.M.; Song, B.; Cao, W.C.; Li, J.; Ye, J. Construction of Highly Water-Stable Metal-Organic Framework UiO-66 Thin-Film Composite Membrane for Dyes and Antibiotics Separation. *Chemical Engineering Journal* **2020**, *385*, 123400, doi:10.1016/j.cej.2019.123400.

12. Wang, J.; Zhang, Y.; Liu, F.; Liu, Y.; Wang, L.; Gao, G. Preparation of a Multifunctional and Multipurpose Chitosan/Cyclodextrin/MIL-68(Al) Foam Column and Examining Its Adsorption Properties for Anionic and Cationic Dyes and Sulfonamides. *ACS Omega* **2023**, *8*, 32017-32026, doi:10.1021/acsomega.3c03897.
13. Wu, S.; Shi, W.; Li, K.; Cai, J.; Xu, C.; Gao, L.; Lu, J.; Ding, F. Chitosan-Based Hollow Nanofiber Membranes with Polyvinylpyrrolidone and Polyvinyl Alcohol for Efficient Removal and Filtration of Organic Dyes and Heavy Metals. *International Journal of Biological Macromolecules* **2023**, *239*, 124264, doi:10.1016/j.ijbiomac.2023.124264.
14. Xu, A.; Gong, Y.; Sun, Q.; Li, L.; Wang, F.; Xiao, Z.; Liu, R. Recoverable Cellulose Composite Adsorbents for Anionic/Cationic Dyes Removal. *International Journal of Biological Macromolecules* **2023**, *238*, 124022, doi:10.1016/j.ijbiomac.2023.124022.
15. Nasri, N.M.; Yusof, E.N.M.; Raman, V.; Ravoof, T.B.S.A.; Rahman, M.B.A.; Abdullah, A.H.; Tahir, M.I.M. New Isostructural ZIFs for Adsorption of Crystal Violet. *Inorganic Chemistry Communications* **2023**, *158*, 111601, doi:10.1016/j.inoche.2023.111601.
16. Tan, Q.; Jia, X.; Dai, R.; Chang, H.; Woo, M.W.; Chen, H. Synthesis of a Novel Magnetically Recyclable Starch-Based Adsorbent for Efficient Adsorption of Crystal Violet Dye. *Separation and Purification Technology* **2023**, *320*, 124157, doi:10.1016/j.seppur.2023.124157.
17. Wang, K.; Zhang, X.J.; Meng, X. Calixpyridinium-Polyoxometalate Nanoparticle Assemblies for the Removal of Organic Dyes from Wastewater. *ACS Appl. Nano Mater.* **2022**, *5*, 13208-13217, doi:10.1021/acsanm.2c02940.
